# Supplementary figures and images for: SMRT and Illumina RNA Sequencing and Characterization of a Key NAC Gene LoNAC29 during the Flower Senescence in Lilium oriental ‘Siberia’
Source: Genes (Basel). 2021 Jun 6;12(6):869. doi: 10.3390/genes12060869 (PMC8227295; doi:10.3390/genes12060869)

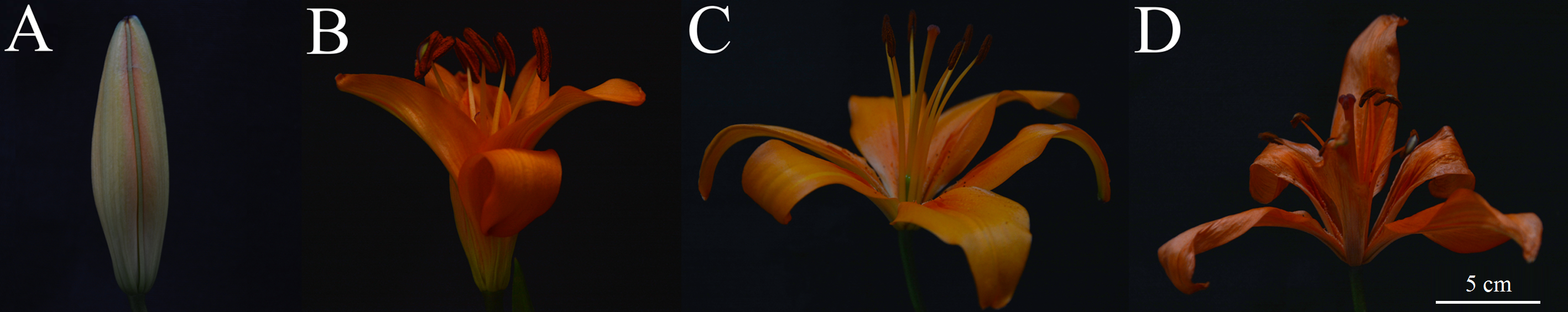

Supplement: Supplementary file 1 [file genes-12-00869-s001.zip › Supplementary data/Figure. S1 Different opening stages of Lilium 'Orange Matrix'.tif]

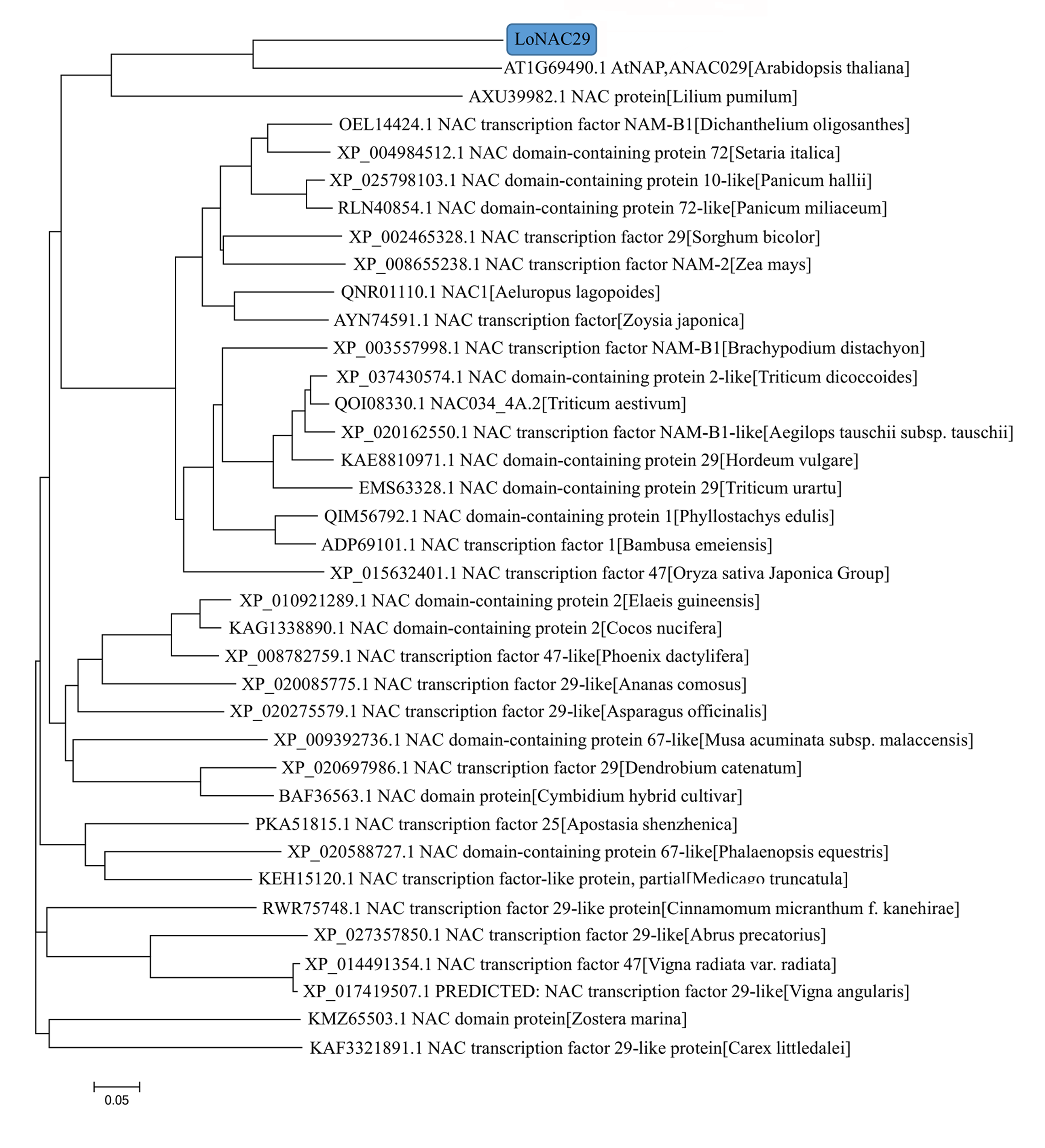

Supplement: Supplementary file 1 [file genes-12-00869-s001.zip › Supplementary data/Figure. S10 Phylogenetic tree of LoNAC29.tif]

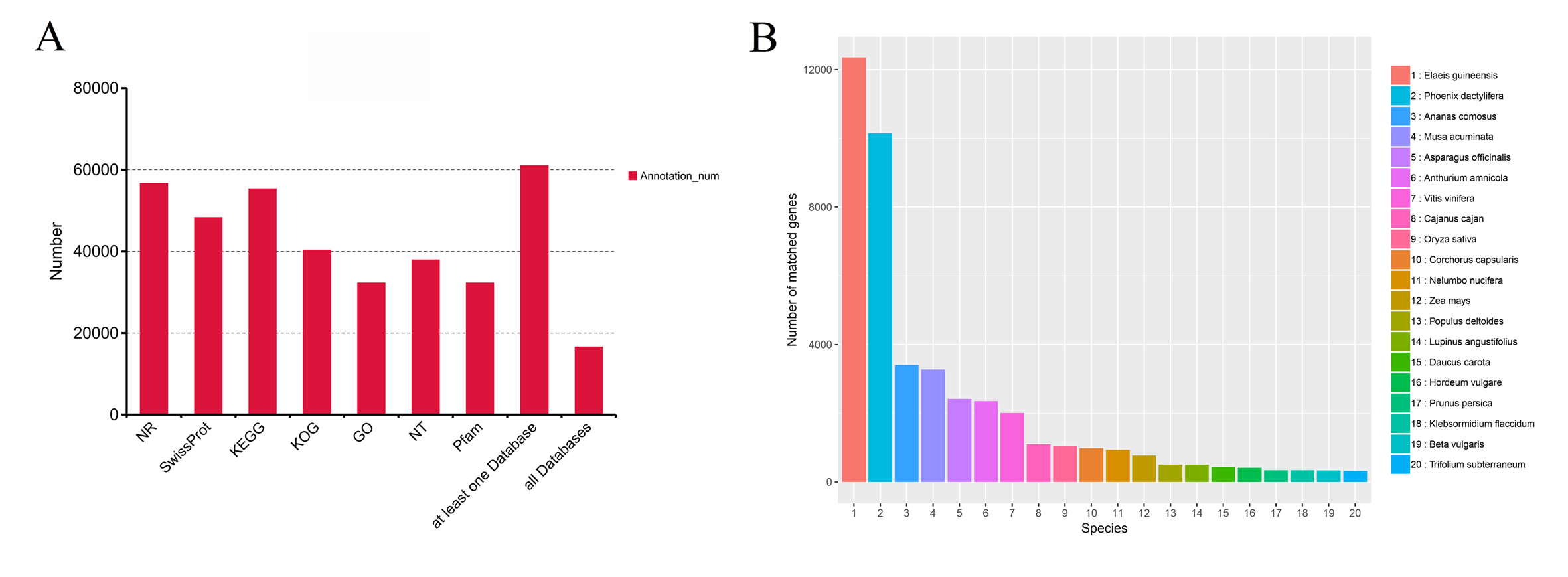

Supplement: Supplementary file 1 [file genes-12-00869-s001.zip › Supplementary data/Figure. S2 Functional annotation.tif]

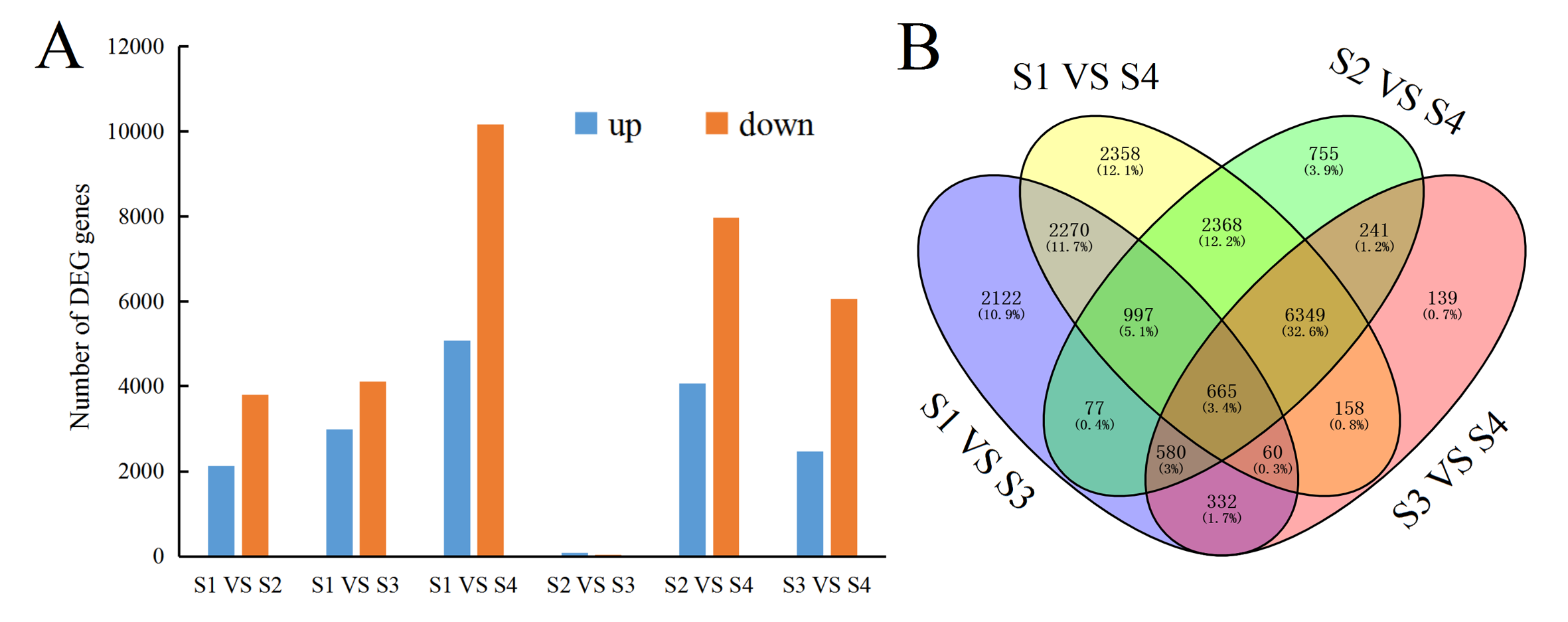

Supplement: Supplementary file 1 [file genes-12-00869-s001.zip › Supplementary data/Figure. S3 Analysis of DEG genes.tif]

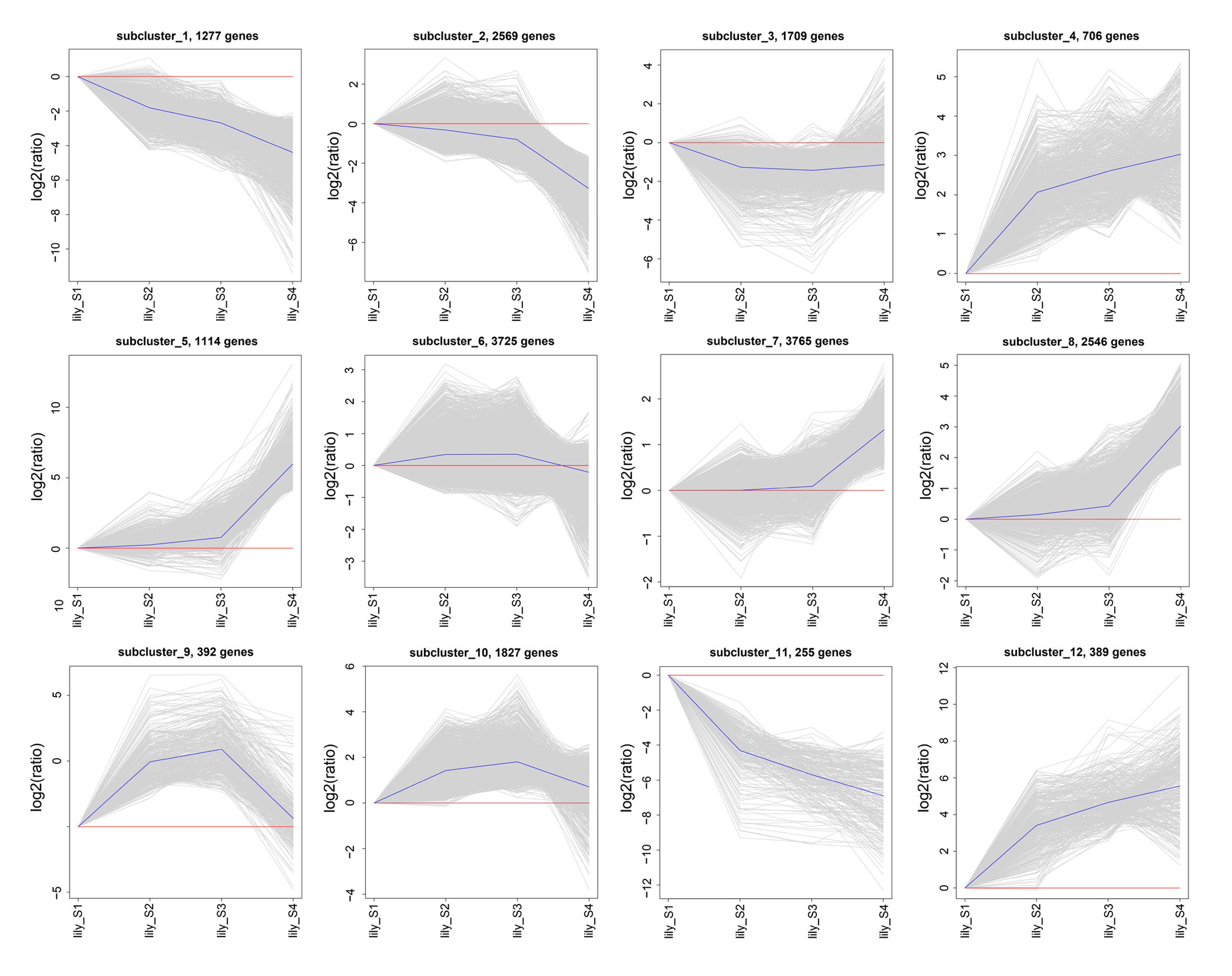

Supplement: Supplementary file 1 [file genes-12-00869-s001.zip › Supplementary data/Figure. S4 Subcluster analysis of differentially expressed genes.tif]

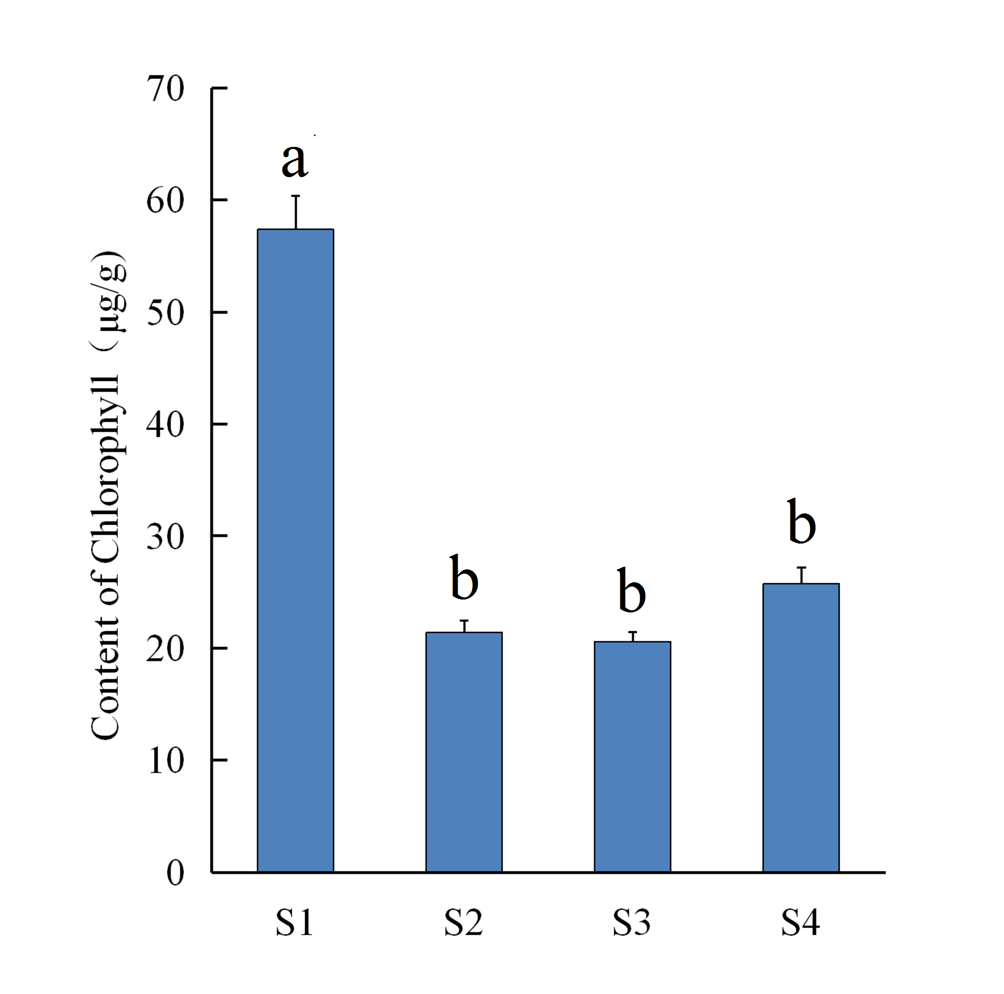

Supplement: Supplementary file 1 [file genes-12-00869-s001.zip › Supplementary data/Figure. S5 Content of chlorophyll during flower opening.tif]

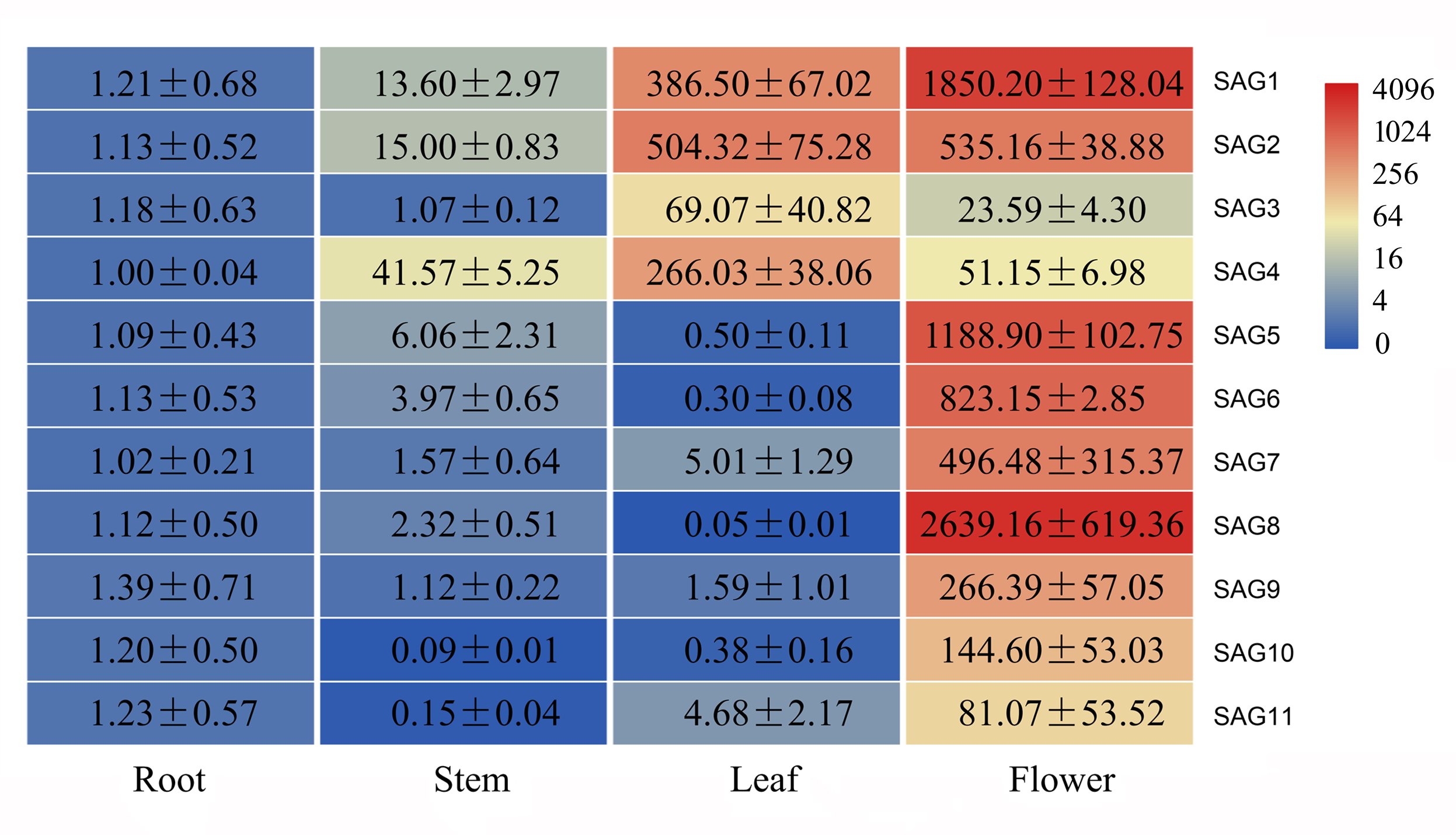

Supplement: Supplementary file 1 [file genes-12-00869-s001.zip › Supplementary data/Figure. S6 Expression of SAG in different organs.tif]

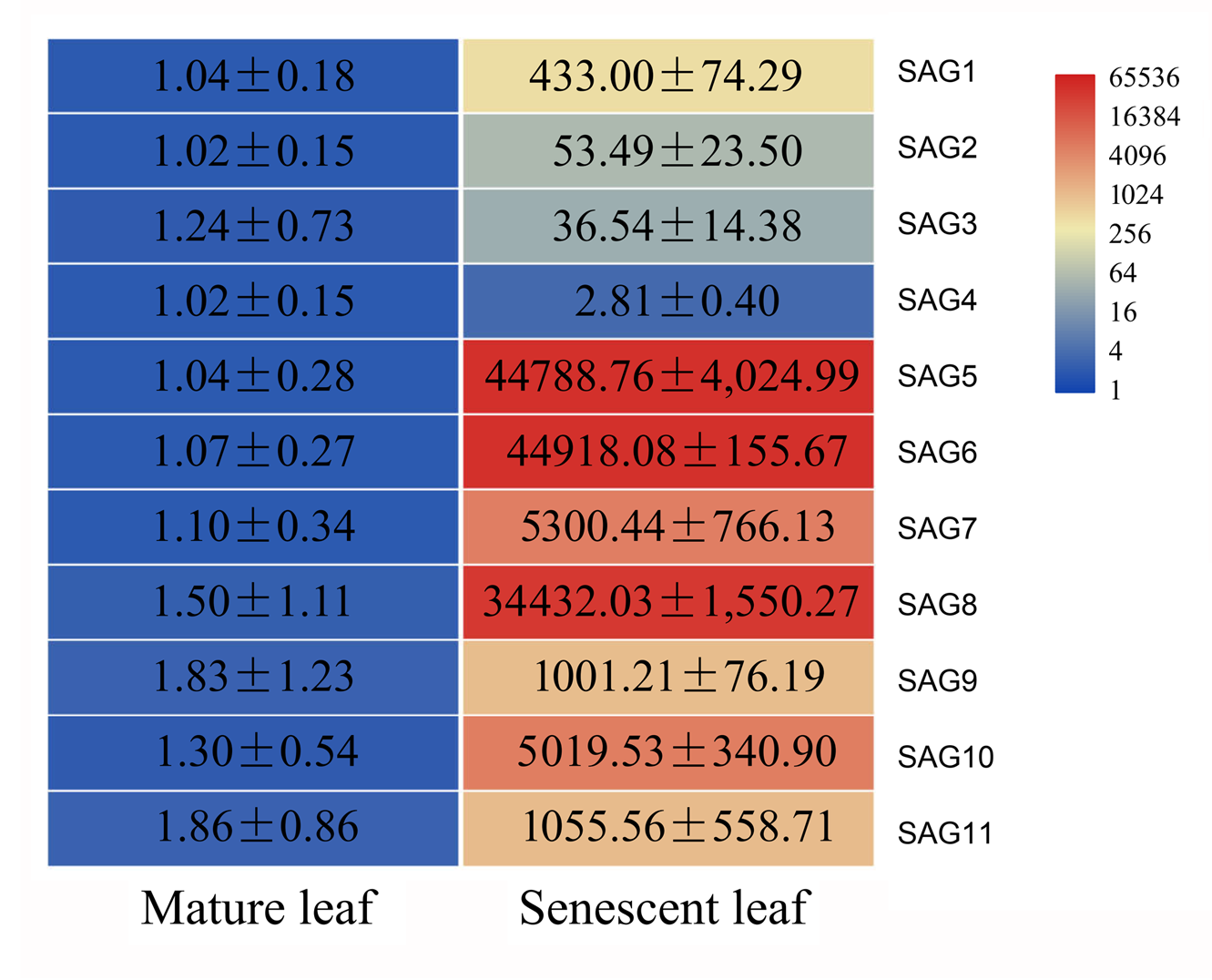

Supplement: Supplementary file 1 [file genes-12-00869-s001.zip › Supplementary data/Figure. S7 Expression of SAG in mature leaf and senescent leaf.tif]

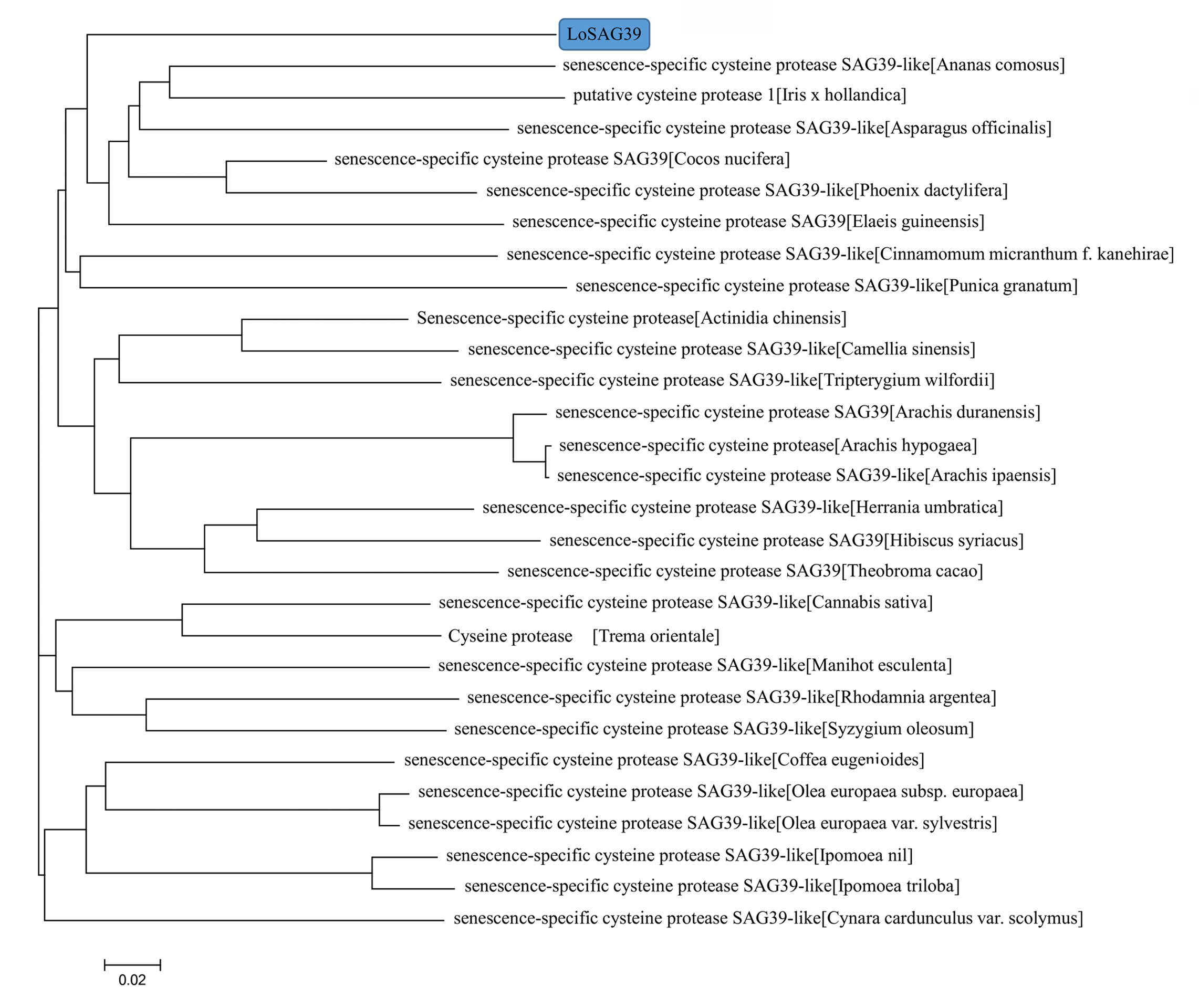

Supplement: Supplementary file 1 [file genes-12-00869-s001.zip › Supplementary data/Figure. S8 Phylogenetic tree of LoSAG39.tif]

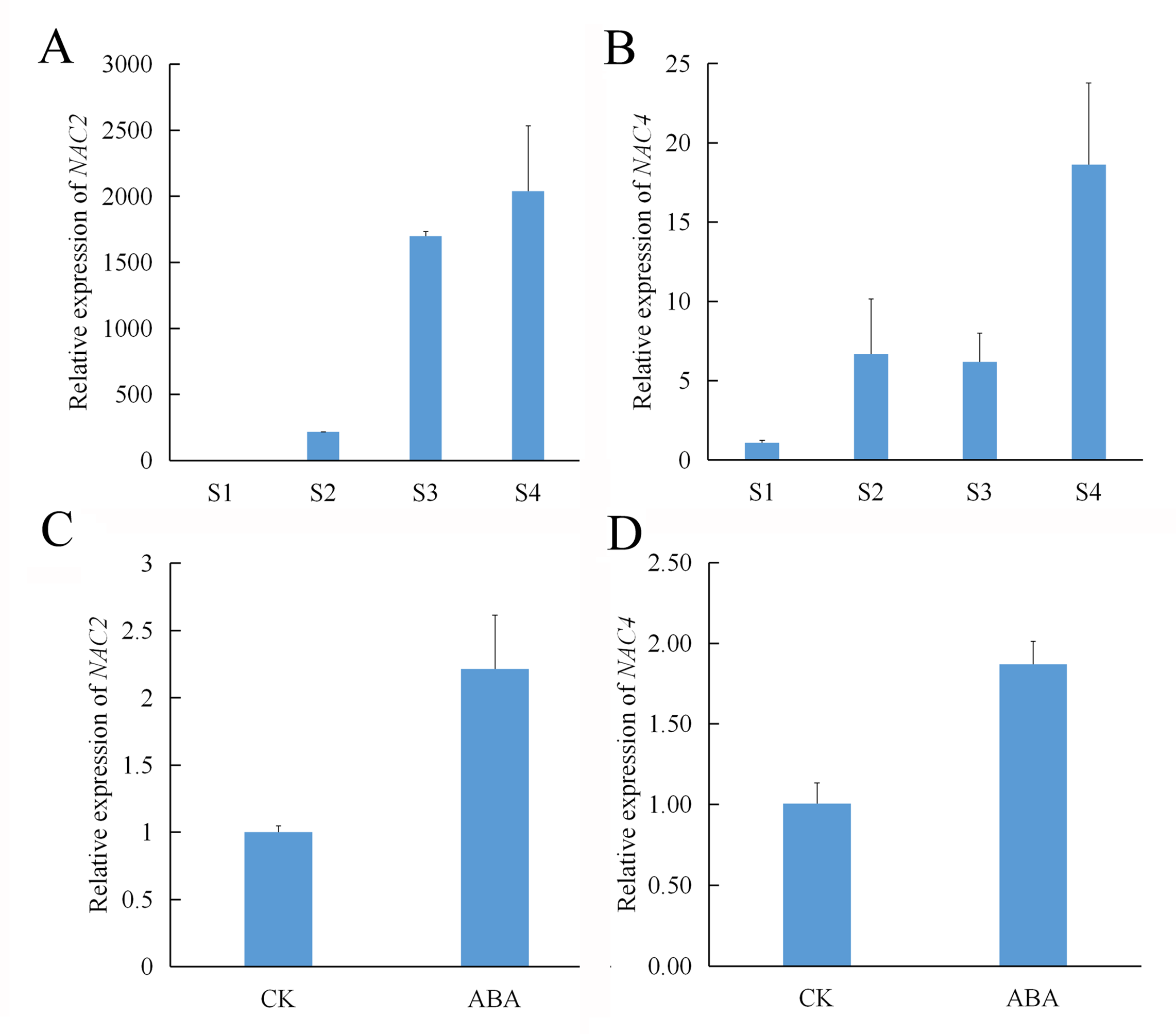

Supplement: Supplementary file 1 [file genes-12-00869-s001.zip › Supplementary data/Figure. S9 Expression of NAC2 and NAC4 in S1-S4 of lily 'Orange Matrix'and ABA treated lily 'Siberia'.tif]
